# Low cyclosporine concentrations in children and time to acute graft versus host disease

**DOI:** 10.1186/s12887-020-02125-6

**Published:** 2020-05-11

**Authors:** Eun Kyung Chung, Jeong Yee, Jae Youn Kim, Hye Sun Gwak

**Affiliations:** 1 Graduate School of Converging Clinical & Public Health, Ewha Womans University, 52 Ewhayeodae-gil, Seodaemun-gu, Seoul, 03760 Korea; 2 Department of Pharmacy, Asan Medical Center, 388-1 Pungnap-dong, Songpa-gu, Seoul, 05535 Korea; 3 College of Pharmacy & Graduate School of Pharmaceutical Sciences, Ewha Womans University, 52 Ewhayeodae-gil, Seodaemun-gu, Seoul, 03760 Korea

**Keywords:** Cyclosporine, Acute graft versus host disease, Allogenic hematopoietic stem cell transplantation, Children

## Abstract

**Background:**

Achievement of target blood concentrations of cyclosporine (CsA) early after transplantation is known to be highly effective for reducing the incidence of acute graft versus host disease (aGVHD). However, no research has been conducted for predicting aGVHD occurrence with low CsA concentrations at different time periods. The objective of this study was to investigate the risk of aGVHD according to low CsA concentrations at lag days in children with allogenic hematopoietic stem cell transplantation (HSCT).

**Methods:**

The records of 61 consecutive children who underwent allogeneic HSCT and received CsA as prophylaxis against aGVHD between May 2012 and March 2015 were retrospectively evaluated. The main outcome was any association between low CsA concentrations at lag days and aGVHD occurrence, which was examined for the first month after transplantation. Mean CsA concentrations at three lag periods were calculated: lag days 0–6, 7–13, and 14–20 before aGVHD occurrence.

**Results:**

Patients whose mean CsA concentrations at lag days 0–6 did not reach the initial target concentration had 11.0-fold (95% confidence interval [CI]: 2.3–51.9) greater incidence of aGVHD. In addition, the AORs of low CsA concentrations at lag days 7–13 and 14–20 for developing aGVHD were 108.2 (95% CI: 7.7–1515.5) and 12.1 (95% CI: 1.1–138.1), respectively.

**Conclusions:**

After low CsA concentrations are detected, careful attention needs to be paid to prevent aGVHD.

## Background

Allogeneic hematopoietic stem cell transplantation (HSCT) is an important treatment method for many hematologic malignancies, bone marrow dysfunctions, immunodeficiency diseases, and metabolic diseases [1, 2]. However, the long-term survival after allogenic HSCT is hindered by the development of human leukocyte antigen and the occurrence of graft-versus-host disease (GVHD). Thus, preventing and treating GVHD are important for reducing morbidity and mortality [3, 4].

Corticosteroids, cyclophosphamide, and antithymocyte globulin have long been used to prevent GVHD, and cyclosporine A (CsA) as an immunosuppressive agent was introduced in the late 1970s. Since then, CsA has been used with methotrexate (MTX) or methylprednisolone. Recently, the combination of CsA and MTX has been used as a standard preventive therapy for acute GVHD (aGVHD) [5, 6].

CsA is an 11 amino acid residue belonging to the group of cyclopeptides isolated from *Tolypocladium inflatum* Gams; it inhibits the early cellular immune response to stimulation and has a T-cell-specific inhibitory effect as well. CsA also binds to a cyclophilin receptor protein to form a heterodimeric complex and inhibits the dephosphorylation of nuclear factor of activated T cells by binding to calcineurin, which acts as a transcription factor for the interleukin-2 gene [7]; in other words, CsA binding to cyclophilin inhibits calcineurin activity and suppresses calcineurin-induced cascade. In addition, CsA increases the expression of transforming growth factor-β, thereby inhibiting the production of cytotoxic T cells and contributing to immunosuppressive activity [8, 9].

Although CsA has been widely used, it is difficult to predict its blood concentrations because of its high pharmacokinetic variability. Moreover, the narrow therapeutic range of CsA requires close monitoring after drug administration [10, 11]. Findings from a number of studies have suggested that low CsA concentrations increase the risk of aGVHD and have shown a correlation between trough CsA concentrations and aGVHD incidence. In particular, researchers have reported that reaching target blood concentrations of CsA early after transplantation is highly effective for lowering the incidence of aGVHD [12–14]; however, there has been no research on predicting aGVHD occurrence with low CsA concentrations at different time periods. Therefore, the purpose of this study was to investigate the risk of aGVHD according to low CsA concentrations at lag days in children who underwent allogenic HSCT.

## Methods

### Study patients

We conducted this retrospective observational study with patients who underwent allogenic HSCT and received CsA from the pediatrics department of Asan Medical Center in Seoul, Korea, from April 2012 to March 2015; we excluded patients with a previous history of transplantation and those who were older than age 18. The study was approved by the Asan Medical Center Institutional Review Board (IRB number: 2017–0509).

The collected data were age, sex, body weight, diagnosis, dates of transplantation and engraftment, use of voriconazole, levels of serum creatinine, aspartate aminotransferase, and alanine aminotransferase, and donor type (sibling, matched unrelated, or mismatched unrelated). We also analyzed the use of busulfan, cyclophosphamide, fludarabine, antithymocyte globulin, and total body irradiation as conditioning regimens and use of MTX and mycophenolate mofetil as concomitant therapy with CsA for preventing aGVHD. In addition, we classified renal function according to the National Cancer Institute Criteria for Adverse Events (NCI CTCAE) based on serum creatinine the day before transplantation.

### CsA administration

CsA was administered intravenously at a rate of 3 mg/kg/day with a 12-h interval from the day before transplantation and was converted to oral dosing after the blood concentration reached a stable target range; patients received an oral dose of CsA (soft capsule) twice daily. We measured trough CsA blood concentrations at least three times per week; the target concentrations were 105–155 ng/mL for patients with sibling donors and 155–210 ng/mL for those with other donor types.

We measured CsA blood concentrations from day 0 to day 30 based on the transplantation day, and the main outcome was any association between low CsA concentrations at lag days and aGVHD occurrence. Mean CsA concentrations at three lag periods were calculated: lag days 0–6, 7–13, and 14–20 before aGVHD occurrence.

### Statistical analysis

We used the chi-squared test or Fisher’s exact test to compare the categorical variables between patients with and without aGVHD and used multivariable logistic regression analysis to identify independent risk factors for aGVHD; multivariate analysis models were constructed using factors with *P* < 0.05 in the univariate analysis along with clinically relevant confounders including sex, age, HLA match and strength of conditioning regimen. We calculated odds ratios and adjusted odds ratios (AOR) from univariate and multivariate analyses, respectively, and considered *P* < 0.05 statistically significant. We performed all statistical analyses using SPSS version 17.0 for Windows (SPSS Inc., Chicago, IL, USA).

## Results

Among 63 eligible patients for this study, we excluded two, one for a previous history of transplantation and one for age > 18. Accordingly, we used the data from 61 pediatric patients for the analysis; Table 1 presents the baseline characteristics of those 61 patients.

**Table 1:** Clinical characteristics of patients (*n* = 61)

Characteristics	No (%) or Mean ± SD	aGVHD No (%) or Mean ± SD		P
		Absence (*n* = 34)	Presence (*n* = 27)	
Age (years)				0.166
< 12	37 (60.7%)	18 (52.9%)	19 (70.4%)	
≥ 12	24 (39.3%)	16 (47.1%)	8 (29.6%)	
Sex				0.980
Female	27 (44.3%)	15 (44.1%)	12 (44.4%)	
Male	34 (55.7%)	19 (55.9%)	15 (55.6%)	
Body weight (kg)	33.8 ± 20.0	37.2 ± 9.5	29.5 ± 20.1	0.138
Diagnosis				0.219
Acute lymphoblastic leukemia	16 (23.2%)	7 (20.6%)	9 (33.3%)	
Acute myeloid leukemia	22 (36.1%)	14 (41.2%)	8 (29.6%)	
Severe plastic anemia	11 (18.0%)	4 (11.8%)	7 (25.9%)	
Myelodysplastic syndromes	7 (11.5%)	6 (17.6%)	1 (3.7%)	
Others	5 (8.2%)	3 (8.8%)	2 (7.4%)	
Donor type				0.331
Sibling	22 (36.1%)	15 (44.1%)	7 (25.9%)	
Mismatched unrelated	13 (21.3%)	6 (17.6%)	7 (25.9%)	
Full matched unrelated	26 (42.6%)	13 (38.2%)	13 (48.1%)	
Conditioning regimen				0.112
Bu/Cy/ATG/Flu^a^	13 (21.3%)	4 (11.8%)	9 (33.3%)	
Bu/Cy/ATG^a^	7 (11.5%)	6 (17.6%)	1 (3.7%)	
Bu/Cy^a^	14 (23.0%)	9 (26.5%)	5 (18.5%)	
Cy/TBI^a^	8 (13.1%)	6 (17.6%)	2 (7.4%)	
Flu/Cy/ATG/TBI	1 (1.6%)	0 (0%)	1 (3.7%)	
Flu/Cy/ATG	10 (16.4%)	4 (11.8%)	6 (22.2%)	
Flu/Cy/TBI	7 (11.5%)	5 (14.7%)	2 (7.4%)	
Cy/ATG	1 (1.6%)	0 (0%)	1 (3.7%)	
Methotrexate				0.041
Yes	48 (78.7%)	30 (88.2%)	18 (66.7%)	
No	13 (21.3%)	4 (11.8%)	9 (33.3%)	
Voriconazole				0.685
Yes	6 (9.8%)	4 (11.8%)	2 (7.4%)	
No	55 (90.2%)	30 (88.2%)	25 (92.6%)	
AST/ALT				0.735
< 200	51 (83.6%)	29 (85.3%)	22 (81.5%)	
≥ 200	10 (16.4%)	5 (14.7%)	5 (18.5%)	
Kidney injury (NCI CTCAE)				0.008
Grade 0–1	50 (82.0%)	32 (94.1%)	18 (66.7%)	
Grade 2–5	11 (18.0%)	2 (5.9%)	9 (33.3%)	
Week reached initial target CsA concentration				0.098
0	2 (3.3%)	2 (5.9%)	0 (0.0%)	
1	16 (26.2%)	10 (29.4%)	6 (22.2%)	
2	33 (54.1%)	20 (58.8%)	13 (48.1%)	
3	7 (11.5%)	1 (2.9%)	6 (22.2%)	
4	3 (4.9%)	1 (2.9%)	2 (7.4%)	
Initial target concentration reached before engraftment				0.155
Yes	44 (72.1%)	27 (79.4%)	17 (63.0%)	
No	17 (27.9%)	7 (20.6%)	10 (37.0%)	
Low CsA concentrations at lag time before aGVHD occurrence				
Lag days 0–6				< 0.001
Yes	28 (45.9%)	8 (23.5%)	20 (74.1%)	
No	33 (54.1%)	26 (76.5%)	7 (25.9%)	
Lag days 7–13				< 0.001
Yes	20 (37.0%)	3 (8.8%)	17 (85.0%)	
No	34 (63.0%)	31 (91.2%)	3 (15.0%)	
Lag days 14–20				0.007
Yes	17 (38.6%)	9 (26.5%)	8 (80.0%)	
No	27 (61.4%)	25 (73.5%)	2 (20.0%)	

^a^Myeloablative conditioning regimen

*aGVHD* acute graft-versus-host disease, *Bu* busulfan, *Cy* cyclophosphamide, *ATG* (rabbit) anti-thymocyteglobulin, *Flu* fludarabine, *TBI* total body irradiation, *AST* aspartate aminotransferase, *ALT* alanine transferase, *NCI CTCAE* National Cancer Institute Common Terminology Criteria for Adverse Events, *CsA* cyclosporine

The median age of the study population was 10.0 years (range: 0.7–18.0), and the median body weight was 31.3 kg (range: 7.4–77.7); 44.3% of patients were female. Acute myelogenous leukemia was the most common disease (22 patients, 36.1%), followed by acute lymphocytic leukemia (16 patients, 23.2%), severe aplastic anemia (11 patients, 18.0%), and myelodysplastic syndrome (7 patients, 11.5%). The donor type proportions were 36.1% siblings, 21.3% mismatched unrelated, and 42.6% matched unrelated, and the median number of engraftment days was 11 (range: 9–22). Twenty-five patients experienced aGVHD within 30 days after transplantation, and the median time to aGVHD occurrence was 11 days (range: 2–30).

The incidence of aGVHD was 0.27 times lower in patients with MTX (*P* = 0.041) and patients without kidney injury (NCI CTCAE grades 2 or higher) had an 8-fold greater incidence of aGVHD (*P* = 0.008). Specifically, patients whose mean CsA concentrations did not reach therapeutic concentrations at lag days 0–6, 7–13, and 14–20 had 9.3, 58.6, and 11.1 times higher, respectively. (Tables 1 and 2).

**Table 2:** Univariate and multivariate logistic regression analysis to identify predictors of acute GVHD related to cyclosporine administration

Characteristics	Unadjusted OR (95% CI)	Adjusted OR (95% CI)		
		Model I	Model II	Model III
Age ≥ 12 (years)	0.474 (0.163–1.375)	1.444 (0.294–7.081)	4.936 (0.413–59.061)	2.447 (0.22–27.203)
Male	0.987 (0.357–2.729)	1.585 (0.389–6.468)	1.626 (0.23–11.471)	1.102 (0.164–7.393)
Mismatched donor	1.633 (0.476–5.600)	1.693 (0.358–7.996)	0.582 (0.04–8.524)	1.479 (0.175–12.480)
Myeloablative conditioning regimen	0.612 (0.206–1.822)	0.376 (0.084–1.692)	0.162 (0.018–1.450)	0.146 (0.022–0.989)^*^
Methotrexate	0.267 (0.072–0.993)^*^	0.264 (0.041–1.721)	0.158 (0.009–2.830)	
Kidney injury grade 0–1 (NCI CTCAE)	8.000 (1.556–41.134)^*^	9.828 (1.434–67.339)^*^	1.800 (0.184–17.596)	
Low CsA concentrations at lag time before aGVHD				
Lag 0–6 days	9.286 (2.882–29.917)^***^	11.017 (2.336–51.947)^**^		
Lag 7–13 days	58.556 (10.632–322.499)^***^		108.196 (7.725–1515.48)^***^	
Lag 14–20 days	11.111 (1.976–62.466)^***^			12.120 (1.064–138.13)^*^

Model I included age, sex, mismatched donor, myeloablative conditioning regimen, methotrexate use, kidney injury (NCI CTCAE grade 2 or higher), and low CsA concentrations at lag days 0–6 before aGVHD. After considering multicollinearity, Model II and III included low CsA concentrations at lag days 7–13 and 14–20 days, respectively

*NCI CTCAE* National Cancer Institute Common Terminology Criteria for Adverse Events, *CsA* cyclosporine, *aGVHD* acute graft-versus-host disease, *OR* odds ratio

^*^P < 0.05, ^**^P < 0.01, ^***^P < 0.001

We constructed multivariate analysis models to determine independent factors for aGVHD occurrence according to low CsA concentrations at lag days 0–6, 7–13, and 14–20. Model I included age, sex, mismatched donors, myeloablative conditioning regimen, MTX use, kidney injury (NCI CTCAE grades 2 or higher) and low CsA concentrations at lag days 0–6, and Models II and III included low CsA concentrations at lag days 7–13 and 14–20, respectively. Patients whose mean CsA concentrations after at lag days 0–6 did not reach the initial targets had 11.0-fold (95% CI 2.3–51.9) greater incidence of aGVHD. The AORs of low CsA concentrations at lag days 7–13 and 14–20 for developing aGVHD were 108.2 (95% CI: 7.7–1515.5, Model II) and 12.1 (95% CI: 1.1–138.1, Model III), respectively. In Model I, patients without kidney injury (NCI CTCAE grades 2 or higher) had 8.0 times greater incidence of aGVHD.

## Discussion

aGVHD is an important complication of allogenic HSCT; authors have reported frequencies of up to 80%. aGVHD occurs by stimulating the immune system of the host, resulting in damage to organs such as the skin, liver, and gastrointestinal tract.

CsA, which is used to prevent aGVHD after a patient receives allogenic HSCT, leads to higher incidence and greater severity of aGVHD in low rather than high doses, but some authors have reported that low doses reduce the recurrence of blood cancer [15, 16]. Therefore, determining the appropriate dose of CsA is important.

CsA blood concentrations can change with lower metabolic rates or depending on CsA excretion rates, which depend on conditions such as renal or liver function. Particularly in pediatric patients, high doses are required to maintain blood concentrations [17] because of their higher distribution volumes and elimination rates compared with those of adults. In addition, appropriate therapeutic concentrations of CsA are affected by conditioning regimens and concomitant medications, and thus there is still much controversy regarding the appropriate treatment concentrations and timing for preventing aGVHD.

Recent study authors have reported that high CsA concentrations within three to 4 weeks after transplantation are more effective in preventing aGVHD. For instance, Garcia et al. [18] reported a correlation between CsA concentration and aGVHD in an adult patient with 156 allogenic HSCTs; low CsA at 3 weeks after transplantation increased the risk of severe aGVHD. Kanda et al. [19] examined the effect of CsA on 171 adult allogenic HSCT patients and found that the CsA concentration within 3 weeks after transplantation was the most important factor in determining the risk of severe aGVHD. On the contrary, other researchers have reported that CsA concentrations in the first 2 weeks after transplantation were significantly related to aGVHD in pediatric transplant recipients, and similarly, in some studies, low CsA concentrations during the first 2 weeks after transplantation in adult transplant recipients increased the risk of aGVHD [12–14].

In contrast to the fact that many study findings suggest that CsA blood concentrations should reach a therapeutic range in the initial stages after transplantation, there is no study of the risk of developing aGVHD based on the lag time after the CsA concentration did not reach the therapeutic range. Although this study had several limitations including heterogeneity of study population and lack of the data on aGVHD severity, to our knowledge, this is the first study to identify the risk of developing aGVHD based on the CsA concentrations at lag time before aGVHD occurrence. Given that one third or more of the study patients did not reach the therapeutic range at one or 2 weeks after transplantation, it is important to predict the occurrence of aGVHD and prepare for it as well as to control the CsA dose.

## Conclusions

We found that the incidence of aGVHD was significantly associated with low CsA concentrations regardless of lag time periods. In particular, we observed the highest associations between incidence of aGVHD and low CsA concentrations at lag days 7–13. Clinicians must pay careful attention to this time periods after they detect low CsA concentrations in order to prevent aGVHD.

## Data Availability

The datasets used and/or analysed during the current study are available from the corresponding author on reasonable request.

## Abbreviations

aGVHD: Acute graft versus host disease
AOR: Adjusted odds ratios
CsA: Cyclosporine
GVHD: Graft-versus-host disease
HSCT: Hematopoietic stem cell transplantation
MTX: Methotrexate
NCI CTCAE: National Cancer Institute Criteria for Adverse Events
